# A New, Non-Invasive Methodology for the Molecular Identification of Adult Sarcophagidae from Collections

**DOI:** 10.3390/insects14070635

**Published:** 2023-07-14

**Authors:** Giorgia Giordani, Daniel Whitmore, Stefano Vanin

**Affiliations:** 1 Dipartimento di Farmacia e Biotecnologie (FABIT), Alma Mater Studiorum Università di Bologna, 40126 Bologna, Italy; giorgia.giordani.gg@gmail.com; 2 Staatliches Museum für Naturkunde Stuttgart, 70191 Stuttgart, Germany; daniel.whitmore@smns-bw.de; 3 Dipartimento di Scienze della Terra dell’Ambiente e della Vita (DISTAV), Università di Genova, 16132 Genova, Italy; 4 National Research Council, Institute for the Study of Anthropic Impact and Sustainability in the Marine Environment (CNR-IAS), 16149 Genova, Italy

**Keywords:** identification, COI, *Sarcophaga*, barcoding, specimen preservation, DNA, forensic entomology

## Abstract

**Simple Summary:**

Species identification is vital in most studies; it can be achieved through the analysis of morphological characters or molecular markers. Unfortunately, DNA extraction involves invasive techniques that lead to the partial or total destruction of specimens, which is not usually acceptable for museum, forensic, and archaeological samples. In this work, a non-invasive DNA extraction technique is described for flies in the genus *Sarcophaga* (Diptera, Sarcophagidae). The technique was tested with successful results on specimens collected between 1889 and 2015.

**Abstract:**

Correct species identification is the cornerstone of all scientific studies that involve insects. Alongside traditional morphological identification techniques, molecular identification based on the characterization and analysis of specific mitochondrial or nuclear gene regions is becoming commonplace. Despite the good results that can be achieved, DNA extraction usually involves invasive techniques that lead to the partial or total destruction of specimens. In this work, a non-invasive DNA extraction technique is described. The technique was tested on the abdomens of dry-preserved Sarcophagidae (Diptera) specimens collected between 1889 and 2015. This allowed for the correct identification of species without impairing diagnostic morphological structures useful for further studies.

## 1. Introduction

More than 1,300,000 species of insects have been described so far; this number makes insects the most abundant taxon of animals, representing about the 75% of all known species. Insects have colonized all terrestrial and freshwater environments on Earth and some species of heteropterans in the genus *Halobates* Eschscholtz 1822 (Heteroptera: Gerridae) are also able to inhabit ocean surfaces. Insects are absent only in the depths of the oceans, where other arthropods such as crabs and shrimps are quite numerous. Insects, due to their large numbers, worldwide distribution, high rate of reproduction, and great adaptability, are common in all anthropic environments in which they can find microhabitats similar to their natural environments or where they can benefit from facultative or obligate associations with humans [1].

In all disciplines—especially in entomology—correct species identification is fundamental to most further considerations [2]. Indeed, developmental rates, food preferences, ecological niches, interactions and relationships with other species, distribution, phenology (seasonality), sensitivity to pathogens, thermal tolerance, and a long list of other parameters are in the vast majority of cases species-specific.

For many years, the evaluation of morphological characters was the only identification method available, and it remains the official and recognized method by which new animal species—including insects—are described and named. The paucity of pictorial materials and the necessity of a specialized taxonomic knowledge in the use of dichotomous identification keys have excluded non-specialists from using this methodology for a long time [3,4]. Furthermore, although some species can be clearly differentiated, others—due to the high similarity of their morphological characteristics—can be discriminated only through an accurate examination of the male or female genitalia [5]. In addition, in some cases, only one sex can be identified with certainty; this happens very often in Diptera, where in some families only males can be clearly discriminated, whereas females often remain unidentified. The lack of specialists for some invertebrate taxa and the need for important revisions of whole families and genera are two other elements that contribute to difficulties in identification. Revisionary studies of genera and families are often complicated, but they remove systematic and taxonomic errors from the literature—as well as problems related to synonymy and old names, which may create a certain confusion among non-specialists. Revisions rely on the work of specialists and on the availability of access to museum collections. Despite some international projects providing support to these activities (e.g., SYNTHESYS, https://www.synthesys.info), the general trend is a global reduction in grants for taxonomic and systematic studies and a general reduction in academic modules dedicated to general taxonomy and species identification. In addition, many regional and local museums are struggling to maintain their historical collections due to a lack of financial support. In addition, taxonomic works are mainly dedicated to the adult stages and only in a few, very specific cases are immature stages considered and described. This lack of attention to the entire developmental cycle and to all the developmental stages of an insect (eggs, larvae, nymphs, puparia, pupae, etc.) can be considered a weakness not only in taxonomic and systematic studies, but also in ecological and faunistic ones. From some points of view, a good knowledge of the morphology of all life stages can help in phylogenetic studies—whereas from an ecological point of view it can provide information on the trophic guild to which the insect belongs, or at least its feeding habits.

In general, most studies are dedicated to taxa that have a direct impact on humans, livestock, and crops, whereas few taxa are considered due mainly to their aesthetic impact—such as their repugnancy (e.g., Heteroptera or true bugs) or beauty (Lepidoptera: butterflies and moths).

In recent years, flies—especially in the families Calliphoridae, Sarcophagidae, and Muscidae—have received special attention due to their role in forensic entomology (FE). Diptera are in fact among the first colonizers of dead bodies, and their development can be used to estimate the minimum time since death or minimum Post-mortem Interval (min PMI). The min PMI corresponds to the time of colonization of a body; depending on the season and the body’s exposure or concealment, colonization may not take place immediately after death. In addition, information concerning the insect’s biology, phenology, distribution, and habitat preferences is used in FE to answer investigative questions concerning the season of death—especially in cold/old cases—and post-mortem body transfer from a primary to a secondary site. Global warming and globalization are affecting the distribution of species, and are also influencing the interpretation of entomological evidence collected from crime scenes [6]. This highlights the need for additional attention in the identification of species, to ensure the recognition and proper identification of allochthonous species. Indeed, southern species are taking advantage of increasing temperatures, and where in the past they were not able to survive because of lower temperatures, they now can survive at cooler latitudes and increase their distribution areas. Cities also represent a special environment with higher temperatures compared to the countryside, providing food sources for flies, other arthropods, and synanthropic vertebrates available throughout the year.

Sarcophagidae flies—commonly known as “flesh flies”, with almost 3000 described species in over 100 genera—are the second-largest family in the dipteran superfamily Oestroidea [7,8]. Members of this family are found almost everywhere, occupying a large diversity of ecological niches and playing a fundamental role in decomposition, pollination, and biocontrol processes. Several species in the genus *Sarcophaga* Meigen, 1824 are useful for min PMI estimation in forensic investigations [9], and some are also involved in myiases. However, the very uniform appearance of members of the genus *Sarcophaga* makes species identification within this genus one of the more challenging obstacles for non-experts, and in several cases only male specimens can be identified with accuracy.

Alongside morphological identification methods, molecular identification based on the characterization and analysis of specific mitochondrial or nuclear regions [2,10,11,12,13,14,15,16,17,18,19,20] has become increasingly frequent in the last 15–20 years. Despite the good results that can be achieved, molecular identification is frequently based on invasive techniques that can lead to the destruction of the specimen, or at least some of its parts (e.g., legs). Various molecular biologists have begun to develop buffers and protocols that extract DNA while preserving the morphological characters of specimens [21]. Indeed, the possibility of preserving the external characters of the sample through morphological and molecular analysis allows re-examination of the material, which is a common need for taxonomists and forensic scientists. Various molecular and morphological works have already been published on Coleoptera from museum collections and archaeological contexts [22,23,24,25,26]. Characters commonly used for taxonomic identification in Diptera—such as chaetotaxy, wing venation, and antennae—are subject to rapid degradation due to their fragility when compared to Coleoptera. In addition, it is worth mentioning that only limited data are available for the morphological and molecular identification of older Diptera specimens [27,28].

DNA extraction by submersion of the whole specimen in a lysis buffer negatively affects the wing structure and the positioning of setae in adult Diptera [27], whereas it generally does not affect diagnostic features in the more robust Coleoptera [22]. A key factor actively conditioning molecular results is time. Successful molecular characterization has been carried out on 20,000-year-old fossil Coleoptera collected from packrat middens [26] and on mosquitoes collected within the past century [28]; for this reason, in this paper, fly specimens collected between 1898 and 2015 were tested.

The possibility of repeating both the molecular and morphological identification of Diptera specimens from contemporary and old cases is important in the forensic field, but also for the preservation of specimens stored in museum collections. The aim of this paper is to present a new methodology for the non-invasive molecular identification of Diptera, based on tests carried out on Sarcophagidae flies. Different extraction methods and specimens with different times since their collection were analyzed to verify which factors have the greatest effect on the amount and quality of the extracted DNA. Sarcophagidae flies in the genus *Sarcophaga* were considered because of their importance in the FE field and because of their peculiar pattern of setae and pruinosity (or microtomentum), which are important diagnostic characters for the identification of species in this family.

## 2. Materials and Methods

### 2.1. Specimen Preparation

Eighty-one (81) dry, adult specimens of sarcophagid flies collected between 1898 and 2015 were tested (Table S1). All specimens belonged to a private collection (S. Vanin) or to the Natural History Museum, London. Two specimens of *Calliphora vomitoria* (Linnaeus, 1758; Diptera, Calliphoridae) and one specimen of *Tenebrio molitor* Linnaeus, 1758 (Coleoptera, Tenebrionidae) from the UK (Huddersfield, West Yorkshire) were also included in the analysis as controls. Control specimens were killed by freezing at −20 °C and then processed as described in the following paragraphs.

**Figure 1:**
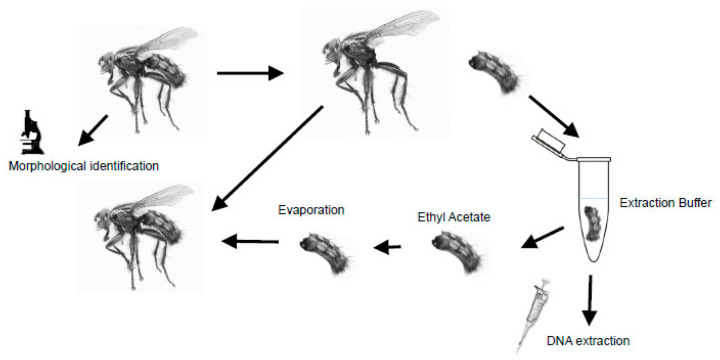
Schematic representation of the identification and extraction protocol.

DNA was extracted from the abdomen of each specimen after it was gently removed from the rest of the body using tweezers and submerged in an extraction solution, as detailed in the next paragraphs (Figure 1). After this first step, each abdomen was dried on absorbent paper, placed for some hours in absolute ethanol to stop further digestion, and passed in ethyl acetate before being re-attached to the rest of the specimen to restore its original appearance (Figure 1). The very fast evaporation of ethyl acetate prevents alterations of the pruinosity and setation. Each specimen was photographed before and after the treatment using a Keyence VHX-S90BE digital microscope. Morphological identification was performed by one of the authors (DW), whereas molecular identification was performed blind by the first author (GG).

### 2.2. DNA Extraction

Five different DNA extraction protocols were tested on the flesh fly abdomens (Table S1):

- Twenty-five specimens were extracted using the QIAamp^®^ DNA Mini kit. DNA was eluted in 200 μL sterile water; half of the abdomens (longitudinally dissected) of three additional specimens were tested with the same commercial kit.
- Fifteen specimens were extracted using the lab-made digestion buffer suggested by Gilbert et al. [22], followed by the QIAquick PCR Purification Kit^®^. DNA was eluted in 40 μL; half of the abdomens of three additional specimens (longitudinally dissected) were tested with the same commercial kit.
- Five specimens were extracted with the homemade digestion buffer proposed by Campos and Gilbert [29], followed by the QIAquick PCR Purification Kit^®^. DNA was eluted in 40 μL.
- Twelve specimens were extracted using the lab-made digestion buffer suggested by Santos et al. [30] and then treated as follows: eight abdomens processed using the QIAquick PCR Purification Kit^®^; two using the QIAamp^®^ DNA Mini kit; and two with the QIAamp^®^ DNA Investigator kit. DNA was eluted in 40 μL for the Purification Kit, 200 μL for the DNA Mini kit, and 100 μL for the Investigator kit.
- Twenty-four specimens were extracted using the QIAamp^®^ DNA Investigator kit. Different elution volumes were used: DNA from 8 specimens was eluted in 100 μL, while for the rest of the samples the elution was performed using 50 μL.

The amount of extracted DNA was quantified using a Qubit^®^ 3.0 Fluorometer, whereas DNA size was evaluated with Agilent Bioanalyzer^®^ 2100.

### 2.3. DNA Amplification

The 658 bp barcode region of the COI gene was targeted with the universal LCO/HCO primers designed by Folmer et al. [31] or through the amplification of smaller, overlapping regions with newly designed primers specific for the genus *Sarcophaga* (Table 1). The total overlapping of the targeted fragments covered the 658 bp region (Figure S1).

Twenty μL of a master mix solution were prepared following the PROMEGA GoTaq^®^ Flexi Polymerase protocol. Four μL of Colourless GoTaq Flexi Buffer (5×), 4 μL of MgCl_2_ (50 mM), 0.5 μL of each primer (10 pmol/μL), 0.5 μL Nucleotide Mix (10 mM), 0.25 μL GoTaq DNA Polymerase (5 u/μL), and 2 to 4 μL of DNA template were used for the amplification of 63 specimens collected in the years 1942–2015 (Table S1). In addition, a PCR master mix plus 1.6 μL BSA (10 mg/mL), 1 μL of each primer (10 pmol/μL), and 1 μL of the nucleotide mix (10 mM) were used for the amplification of another 17 specimens collected in the years 1898–2004, to reduce the potential inhibitory effects of the exogenous molecules co-extracted with the DNA. For these last reactions, up to 5 μL of DNA per reaction were used.

PCR was performed with an initial heat activation step at 95 °C for 10 min; 35 cycles at 95 °C for 1 min, 47 °C for 1 min, 72 °C for 1 min, and a final extension step at 72 °C for 10 min, set up on a BioRad C1000 Thermal Cycler (Bio-Rad Laboratories, Inc., Watford, UK).

Each reaction was visualized on 1.5% agarose gel previously stained with Midori Green Advanced DNA Stain (Geneflow, Elmhurst, UK).

Samples that showed a clear band of the appropriate molecular weight were purified using the QIAquick PCR Purification Kit^®^ (QIAGEN), following the manufacturer’s instructions. Purified amplicons were eluted in 40 μL of sterile/deionized water and sequenced by an external company (Eurofins Operon MWG, Ebersberg, Germany), using a one-directional sequencing process based on the standard Sanger method.

### 2.4. Bioinformatics and Statistics

EMBOSS Merger [32] was used to reconstruct the COI gene sequences; sequences obtained from amplification with the newly-designed primers were aligned in pairs to find the overlap points and recreate the consensus sequence of the COI gene. A manual check of the chromatograms was performed to verify the goodness of the concatenated sequence.

The online system BLASTn^®^ [33], provided by NCBI, was used for species identification based on a percent match with those available in online gene banks.

All statistical analyses were performed using the IBM SPSS 22.0 software (IBM SPSS Statistics, Armonk, NY, USA). The significance threshold was set at 5%.

## 3. Results and Discussion

### 3.1. Morphological Identification

All morphological characters present on the head and thorax were not affected by the methods described here, as DNA was extracted only from the flies’ abdomens.

Preserving the features of the head plays an important role in identification—not only at the species level but also at the family level. The inclination (convergent, parallel, or divergent) of the post-ocellar bristles, for example, is vital for distinguishing Heleomyzidae and Piophilidae (postvertical bristles are convergent or sometimes crossed in Heleomyzidae, and divergent, parallel, or absent in Piophilidae). The setation of the arista is another well-established character used in the identification of Sarcophagidae, whereas the pruinosity of the facial area is commonly used in several other families.

It is common to use fly legs for DNA extraction, to reduce the alteration of specimens; however, the position of the setae on the tibiae and femora is commonly used in several families for the identification of species not only of forensic interest, such as in Calliphoridae (e.g., *Melanomya* vs. *Angioneura*) and Syrphidae (*Platycheirus* spp.) [34]. Furthermore, the presence of setae on the coxae can be diagnostic when distinguishing between *Lucilia* and *Chrysomya* among the Calliphoridae [35]. The distribution of setae on the femora is also important in the genus *Sarcophaga*—for example, in distinguishing *Sarcophaga* (*Helicophagella*) *crassimargo* from *Sarcophaga* (*Helicophagella*) *hirticrus* [36].

Thoracic and wing characteristics are also particularly important. In addition to the pruinosity and color of the thorax, the distribution of setae on the thorax and scutellum (e.g., marginal) play an important role in species identification. For example, *Sarcophaga melanura*, *S. variegata,* and *S. crassipalpis* show different distribution patterns of the postsutural dorsocentral bristles, both in terms of number and position.

**Figure 2:**
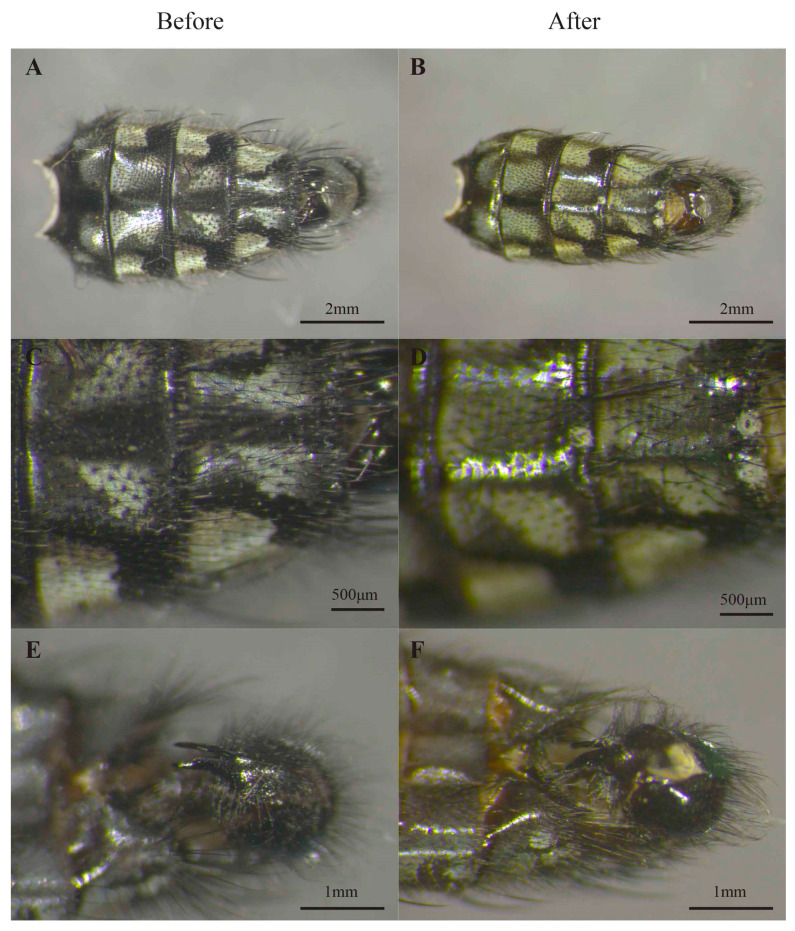
Morphological characteristics before (**A**,**C**,**E**) and after (**B**,**D**,**F**) DNA extraction: (**A**,**B**) abdomen; (**C**,**D**) checkerboard pattern; (**E**,**F**) male genitalia.

Diagnostic characteristics such as the checkerboard pattern of pruinosity on the abdomen or the genital structures were conserved during DNA extraction—as shown in Figure 2. Although some hairs/setae were lost after the extraction process, their original positions could still be detected by observing the position of their alveoli, which are easily visible at the appropriate magnification.

Male [37] and female [20] genitalia play a key role for species identification in Diptera. The shape and size—as well as the distribution of setae—on the genitalia are robust diagnostic characters. As illustrated in Figure 2E,F, the genitalia and their setation were not affected by the extraction methods, and further preparation and mounting of these structures could still be performed if needed. Indeed, in some cases, the genitalia must be diaphanized in a NaOH/KOH solution and then mounted on a microscope slide in order to visualize their tiniest morphological details.

### 3.2. DNA Extraction

Five different DNA extraction protocols were tested on the flesh fly abdomens: all of them provided positive results, as reported in Table S1.

### 3.3. DNA Amplification

**Figure 3:**
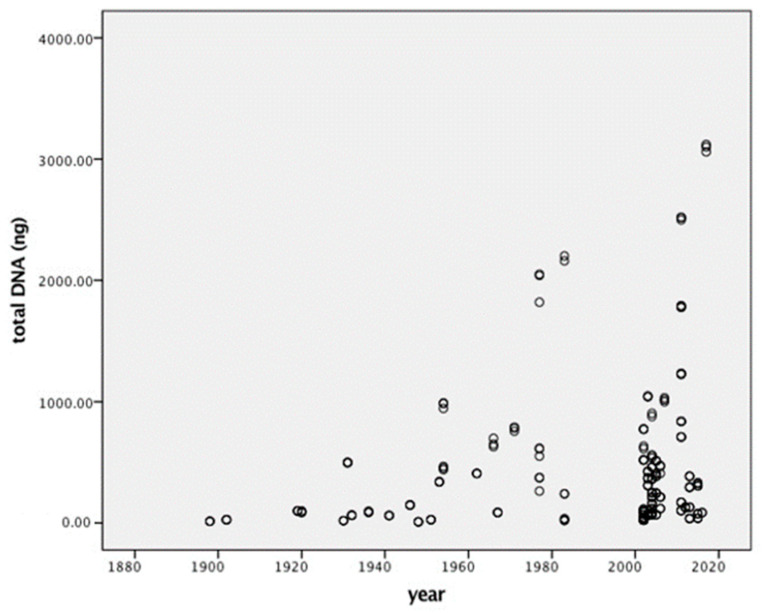
Total DNA extracted from *Sarcophaga* specimens collected between 1898 and 2015. The graph shows a decrease in the maximum amount of DNA extracted depending on time since collection; however, it also shows a high degree of variability that could be associated with the killing methods and storage conditions undergone by the specimens. This information, however, was not available to us.

The average DNA yield for samples collected in the 1980s was 619 ± 906 ng, in the 1970s 918 ± 630 ng, in the 1960s 372 ± 286 ng, in the 1950s 448 ± 341 ng, in the 1940s 73 ± 57 ng, in the 1930s 168 ± 192 ng, and in the 1910–1930s 96 ± 6 ng. The two oldest samples, collected in 1902 and 1898, allowed the recovery of 26 ± 1 and 13 ± 1 ng of DNA, respectively. The high variability in the amount of DNA extracted from specimens collected in the same year underlines how a positive extraction result could be correlated to the size of the specimen, its history, and its collection method. Unfortunately, this kind of information was not reported on the insects’ labels and could not be derived from other sources, except in few cases where detailed reports written by the collectors existed. A statistical analysis was carried out to understand the relationship between the total DNA extracted and time since collection. Despite the presence of a trend (Figure 3), the correlation coefficient was not robust (r = 0.182). A two-way ANOVA was used to verify the effect of time since collection (with specimens grouped by 10-year intervals), of the extraction method, and of the interaction between these two factors and the amount of DNA extracted. Whereas the influence of the extraction method was not statistically significant (F = 1.651, df = 7, *p* = 0.123), time since collection had a significant impact on DNA yield (F = 5.072, df = 12, *p* = 0.000). The interaction between the two parameters (years*extraction kit) did not have any significant effect (F = 1.833, df = 7, *p* = 0.083).

Three *Sarcophaga* abdomens (samples 39–41) were split into two and tested in parallel for two extraction methods (Homemade Digestion Buffer by Gilbert et al. [20] followed by QIAquick PCR Purification Kit^®^ and QIAamp^®^ DNA Mini kit) for a reliable comparison of these methods. Quantification of the extracted DNA (Table S1) from the same specimen showed a better efficacy with the Homemade Digestion Buffer compared to the commercial kit.

### 3.4. DNA Amplification and Sequence Analysis

PCR amplification targeted the barcode region of the mitochondrial gene COI. Amplification with the universal primer LCO/HCO [31] gave positive results only for fresh samples (2015–2017; data not shown). The newly-designed primers (Table 1) allowed for the amplification of around 200 bp for a high number of samples (Table S2). Due to the dominant presence of the A + T composition—which is characteristic of insect DNA [38]—and the presence of some inner repetitions, amplification of the COI barcode portion with the 411 FW and RV primers (Table S2) was often problematic, producing weak results.

Molecular identification of single fragments and of the reconstructed COI barcode region was carried out in BLASTn^®^ [32]. The positive amplification of a 151 bp fragment allowed for correct identification at the species level of a 98-year-old specimen. Amplification of older specimens allowed for identification only at the genus level.

From among 80 sequences of around 200 bp of *S. variegata*, 70 resulted in a positive identification at the species level. Among these, 59% shared the same identity percentage and score with *S. lehmanni*. The use of the concatenated sequence, of around 700 bp, decreased this percentage to 7%. This result is in agreement with Jordaens et al. [39], according to whom a 200 bp fragment is not sufficient for species identification in Sarcophagidae. On the contrary, 20 short sequences of *S. lehmanni* resulted in 17 positive identifications at the species level, of which 75% shared the same identity percentage and score with *S. variegata*. The concatenated gene did not give any positive identification result. From among 43 positive identifications of around 200 bp sequences of *S. carnaria*, 100% showed the same identity percentage and score with *S. pyrenaica*. The concatenated gene decreased this percentage to only 79%.

## 4. Conclusions

This study examined a non-invasive method for the identification of flesh fly specimens through their mitochondrial DNA. From a forensic and a museum collection perspective, maintaining the physical integrity of entomological samples is crucial. In fact, it allows for the re-examination of specimens both for systematic studies and for forensic investigations, especially when dealing with min PMI estimation. It is also worth mentioning that *Sarcophaga* flies can be involved in myiasis. In cases of nosocomial myiases (myiases occurring in hospitals and other care structures), it is particularly important to identify the potential responsibilities of medical staff and other caregivers, and the correct identification of *Sarcophaga* species can help detect potential cases of neglect—for example of children, elderly, or bedridden patients.

In the literature, previous studies have described non-destructive extraction methods for insect specimens [22,40,41]. However, these works have dealt mainly with beetles—which have fairly robust exoskeletons—or with fresh samples. Based on our results, the non-destructive molecular analysis of Sarcophagidae abdomens allows for the quite accurate identification of specimens without impairing the delicate morphological structures useful for further studies. DNA extraction and amplification cannot necessarily be expected from all specimens; indeed, a variation in the collection and storage conditions of flies may adulterate the DNA survival rate—as already suggested by Gilbert et al. [22] for beetles and confirmed here.

Additionally, our method did not affect the pruinosity (microtomentum) of the abdomen and the shape and structure of the genitalia (as well as their setation) of the analyzed specimens. These characters are diagnostic for species identification in the genus *Sarcophaga* but are also important in other dipteran taxa.

Further research on other taxa and using methods allowing for a faster evaporation of the solvent will be carried out in future, providing non-invasive techniques for studying precious specimens preserved in museum collections.

## Figures and Tables

**Table 1:** Newly designed degenerated primers for the genus *Sarcophaga*.

Oligo Name	Sequence (5′ -> 3′)	Tm (°C)
Sar111_FW	TCGCAACAATGGTTATTCTCT	54.0
Sar111_RV	TCARTTTCCAAAYCCTCCAAT	54.2
Sar211_FW	GTAATTGTTACAGCYCATGC	54.2
Sar211_RV	TTCCAGCTCCRTTTTCTACT	54.0
Sar311_FW	CYCGAATRAAYAATATAAGTTTTTG	56.4
Sar311_RV	CCTAAAATTGAAGAAATTCCWGCTA	60.3
Sar411_FW	CTAATATTGCYCATGGRGGAGC	58.5
Sar411_RV	CGRTCAGTTAATARTATRGTRATWGC	50.8
Sar511_FW	GGWATTACHTTTGAYCGAAT	54.7
Sar511_RV	GAYTCTTGRCTAATAATGTGAG	52.9

## Data Availability

All specimens belong to a private collection (S. Vanin) or to the Natural History Museum, London and are available for additional study on request.

## Supplementary Materials

The following supporting information can be downloaded at: https://www.mdpi.com/article/10.3390/insects14070635/s1, Figure S1: Primer scheme for concatenated sequences; Table S1: List of specimens used in the extraction experiment; Table S2: Results of the molecular identifications.
