# Supplementary material for: A New, Non-Invasive Methodology for the Molecular Identification of Adult Sarcophagidae from Collections
Source: Insects. 2023 Jul 14;14(7):635. doi: 10.3390/insects14070635 (PMC10380211; doi:10.3390/insects14070635)
Supplement: Supplementary file 1 [file insects-14-00635-s001.zip › insects-2445916-supplementary.pdf]

Supplementary Materials

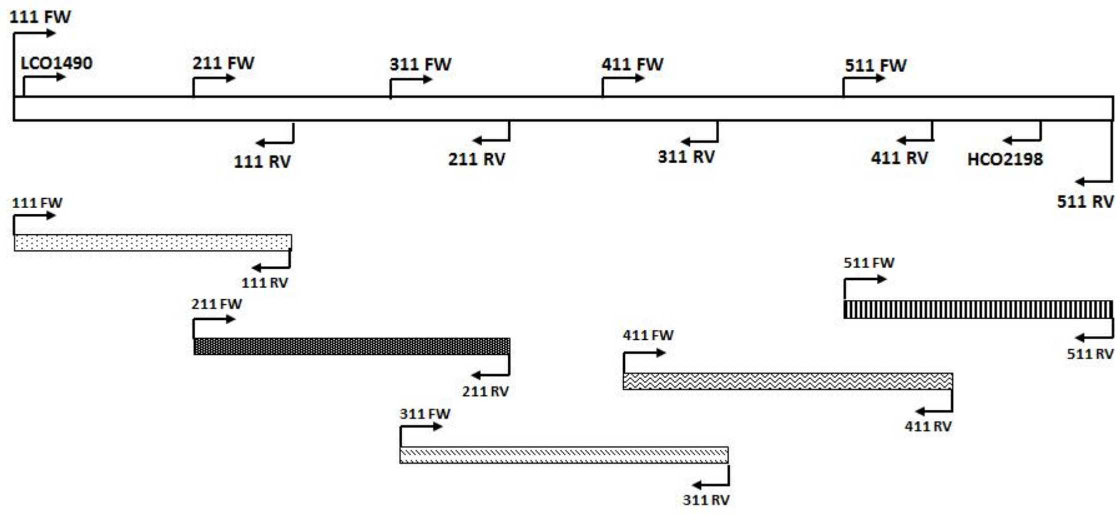

Figure S1: Primer scheme for concatenated sequences. The whole sequence length corresponds to 658bp of the COI mitochondrial gene.

Table S1. List of specimens used in the extraction experiment. The year of collection, the extraction method used, the morphological identification and the average amount of extracted DNA ± St.dev (ng/µl) are reported. * refers to half specimens. Orange lines refer to control samples.

Sample	Year	Extraction method	Morphological ID	Average DNA quantification (ng)
1	2015	QIAamp® DNA Mini kit	*S. variegata*	328.00±3.46
2	2011	QIAamp® DNA Mini kit	*S. crassipalpis*	102.13±0.61
3	2015	QIAamp® DNA Mini kit	*S. baranoffi*	39.20±0.40
4	2011	QIAamp® DNA Mini kit	*S. crassipalpis*	708.00±0.00
5	2015	QIAamp® DNA Mini kit	*C. vomitoria*	76.40±0.40
6	2011	QIAamp® DNA Mini kit	*S. crassipalpis*	2513.33±11.55
7	2011	QIAamp® DNA Mini kit	*S. crassipalpis*	837.33±2.31
8	2002	QIAamp® DNA Mini kit	*S. variegata*	45.33±0.46
9	2013	QIAamp® DNA Mini kit	*S. variegata*	36.67±0.23
10	2013	QIAamp® DNA Mini kit	*S. variegata*	130.27±0.46
11	2004	QIAamp® DNA Mini kit	*S. variegata*	82.80±0.00
12	2003	QIAamp® DNA Mini kit	*S. carnaria*	60.40±0.00
13	2004	QIAamp® DNA Mini kit	*S. carnaria*	160.27±0.23
14	2002	QIAamp® DNA Mini kit	*S. emdeni*	24.53±0.23
15	2002	QIAamp® DNA Mini kit	*S. lehmanni*	772.00±0.00
16	2002	DB Gilbert et al (2007) + QIAquick PCR Purification Kit®	*S. variegata*	97.07±0.46
17	2002	DB Gilbert et al (2007) + QIAquick PCR Purification Kit®	*S. variegata*	518.67±2.31
18	2002	DB Gilbert et al (2007) + QIAquick PCR Purification Kit®	*S. variegata*	101.60±0.00
19	2003	DB Gilbert et al (2007) + QIAquick PCR Purification Kit®	*S. variegata*	309.33±0.46
20	2003	DB Gilbert et al (2007) + QIAquick PCR Purification Kit®	*S. lehmanni*	424.00±0.00

21	2006	DB Gilbert et al (2007) + QIAquick PCR Purification Kit®	*S. carnaria*	213.60±0.80
22	2007	DB Gilbert et al (2007) + QIAquick PCR Purification Kit®	*S. croatica*	1016.00±16.00
23	2005	DB Gilbert et al (2007) + QIAquick PCR Purification Kit®	*S. croatica*	406.67±2.31
24	2015	DB Gilbert et al (2007) + QIAquick PCR Purification Kit®	*S. variegata*	306.40±0.80
25	2013	DB Gilbert et al (2007) + QIAquick PCR Purification Kit®	*S. variegata*	294.40±3.20
26	2013	DB Gilbert et al (2007) + QIAquick PCR Purification Kit®	*S. variegata*	384.80±0.80
27	2012	DB Gilbert et al (2007) + QIAquick PCR Purification Kit®	*C. vomitoria*	128.80±0.80
28	2011	DB Gilbert et al (2007) + QIAquick PCR Purification Kit®	*S. crassipalpis*	1784.00±8.00
29	2011	DB Gilbert et al (2007) + QIAquick PCR Purification Kit®	*S. crassipalpis*	1229.33±4.62
30	2011	DB Gilbert et al (2007) + QIAquick PCR Purification Kit®	*S. crassipalpis*	169.87±0.46
31	2003	QIAamp® DNA Mini kit	*S. crassipalpis*	1044.00±4.00
32	2004	QIAamp® DNA Mini kit	*S. crassipalpis*	212.00±2.00
33	2004	QIAamp® DNA Mini kit	*S. crassipalpis*	363.33±3.06
34	2004	QIAamp® DNA Mini kit	*S. crassipalpis*	116.80±0.40
35	2004	QIAamp® DNA Mini kit	*S. crassipalpis*	460.00±0.00
36	2004	QIAamp® DNA Mini kit	*S. argyrostoma*	248.00±2.00
37	2003	QIAamp® DNA Mini kit	*S. argyrostoma*	368.67±5.03
38	2017	QIAamp® DNA Mini kit	*T. molitor*	3093.33±30.55
39a*	2006	DB Gilbert et al (2007) + QIAquick PCR Purification Kit®	*S. croatica*	469.00±5.66
39b*	2006	QIAamp® DNA Mini kit	*S. croatica*	385.50±3.54
40a*	2005	DB Gilbert et al (2007) + QIAquick PCR Purification Kit®	*S. croatica*	85.50±0.71
40b*	2005	QIAamp® DNA Mini kit	*S. croatica*	118.60±0.85
41a*	2016	DB Gilbert et al (2007) + QIAquick PCR Purification Kit®	*S. carnaria*	68.00±1.13
41b*	2016	QIAamp® DNA Mini kit	*S. carnaria*	38.00±1.13
42	2002	QIAamp® DNA Mini kit	*S. variegata*	91.60±1.70
43	2003	QIAamp® DNA Mini kit	*S. lehmanni*	70.80±6.22
44	2002	QIAamp® DNA Investigator kit	*S. variegata*	114.00±1.41
45	2002	QIAamp® DNA Investigator kit	*S. lehmanni*	622.00±14.14
46	2002	DB Santos et al (2018) + QIAquick PCR Purification Kit®	*S. croatica*	22.96±0.45
47	2002	DB Santos et al (2018) + QIAquick PCR Purification Kit®	*S. variegata*	43.20±0.00
48	2002	DB Santos et al (2018) + QIAamp® DNA Mini kit	*S. variegata*	23.20±2.83
49	2002	DB Santos et al (2018) + QIAamp® DNA Mini kit	*S. croatica*	36.80±0.00
50	2002	DB Santos et al (2018) + QIAamp® DNA Investigator kit	*S. variegata*	22.60±0.00
51	2004	DB Santos et al (2018) + QIAamp® DNA Investigator kit	*S. variegata*	67.20±0.00
52	2004	DB Santos et al (2018) + QIAquick PCR Purification Kit®	*S. variegata*	892.00±16.97
53	2002	DB Santos et al (2018) + QIAquick PCR Purification Kit®	*S. variegata*	36.36±1.19
54	1983	QIAamp® DNA Investigator kit	*S. carnaria*	2180.00±28.28

55	1983	QIAamp® DNA Investigator kit	*S. subvicina*	240.00±0.00
56	1983	DB Santos et al (2018) + QIAquick PCR Purification Kit®	*S. carnaria*	20.56±0.11
57	1983	DB Santos et al (2018) + QIAquick PCR Purification Kit®	*S. subvicina*	34.12±0.40
58	2002	DB Santos et al (2018) + QIAquick PCR Purification Kit®	*S. variegata*	30.00±6.34
59	2002	DB Santos et al (2018) + QIAquick PCR Purification Kit®	*S. lehmanni*	70.00±1.13
60	1967	QIAamp® DNA Investigator kit	*S. carnaria*	86.30±0.85
61	1966	QIAamp® DNA Investigator kit	*S. subvicina*	657.33±34.95
62	1946	QIAamp® DNA Investigator kit	*S. carnaria*	147.67±2.08
63	1942	QIAamp® DNA Investigator kit	*S. carnaria*	61.20±0.72
64	2004	Campos and Gilbert 2012 + ppK	*S. carnaria*	550.00±12.49
65	2005	Campos and Gilbert 2012 + ppK	*S. croatica*	511.33±1.15
66	2005	Campos and Gilbert 2012 + ppK	*S. croatica*	245.33±2.31
67	1962	Campos and Gilbert 2012 + ppK	*S. carnaria*	407.67±0.58
68	1948	Campos and Gilbert 2012 + ppK	*S. carnaria*	9.37±0.21
69	1977	QIAamp® DNA Investigator kit	*S. carnaria*	591.67±36.17
70	1977	QIAamp® DNA Investigator kit	*S. carnaria*	336.33±63.54
71	1977	QIAamp® DNA Investigator kit	*S. carnaria*	1970.00±130.00
72	1971	QIAamp® DNA Investigator kit	*S. carnaria*	775.00±18.03
73	1954	QIAamp® DNA Investigator kit	*S. carnaria*	452.67±13.01
74	1954	QIAamp® DNA Investigator kit	*S. carnaria*	973.33±24.66
75	1953	QIAamp® DNA Investigator kit	*S. carnaria*	338.67±2.08
76	1951	QIAamp® DNA Investigator kit	*S. carnaria*	26.93±0.25
77	1932	QIAamp® DNA Investigator kit	*S. carnaria*	63.50±2.60
78	1931	QIAamp® DNA Investigator kit	*S. carnaria*	497.33±3.06
79	1930	QIAamp® DNA Investigator kit	*S. carnaria*	19.13±0.15
80	1936	QIAamp® DNA Investigator kit	*S. carnaria*	90.83±5.77
81	1920	QIAamp® DNA Investigator kit	*S. carnaria*	92.00±7.76
82	1919	QIAamp® DNA Investigator kit	*S. carnaria*	99.67±0.29
83	1902	QIAamp® DNA Investigator kit	*S. subvicina*	26.40±1.04
84	1898	QIAamp® DNA Investigator kit	*S. subvicina*	13.47±0.61

Table S2. Results of the molecular identifications. Legend: ✓✓ (Bright green): Correct identification at species level; ✓✓* (Dark green): Correct identification at species level but same score and identity percentage with other species; ✓ (Yellow): Correct identification at genus level; - (Red): positive PCR amplification but wrong identification; X (Grey): negative PCR amplification. Empty boxes indicate reactions that were not performed.

**Sample**	**Year**	**Sar111**	**Sar211**	**Sar311**	**Sar411**	**Sar511**	**Concatenated gene**	**Morphological ID**
**1**	2015	✓✓	✓✓*	✓✓	✓✓*	✓✓*	*S.variegata*	*S.variegata*
**2**	2011	✓	✓	✓✓	✓✓	✓✓	*S.crassipalpis*	*S.crassipalpis*
**3**	2015	✓	✓	✓	✓	✓	*S.subvicina*	*S.baranoffi*
**4**	2011	✓	✓✓	✓✓	✓✓	✓✓	*S.crassipalpis*	*S.crassipalpis*
**6**	2011	✓✓	✓✓	✓✓	✓✓	✓✓	*S.crassipalpis*	*S.crassipalpis*
**7**	2011	✓✓	✓✓	✓✓	✓✓	✓✓	*S.crassipalpis*	*S.crassipalpis*
**8**	2002	✓✓*	✓✓*	-	-	-	*C.vomitoria*	*S.variegata*
**9**	2013	✓✓	✓✓*	✓✓	✓✓*	✓✓*	*S.variegata*	*S.variegata*
**10**	2013	✓✓*	✓✓*	✓✓	✓✓	✓✓*	*S.variegata*	*S.variegata*
**11**	2004	✓✓*	✓✓	-	✓✓*	✓✓*	*S.variegata*	*S.variegata*
**12**	2003	✓✓*	✓✓*	✓	✓✓*	✓✓*	*S. carnaria*	*S. carnaria*

**13**	2004	✓✓*	✓✓*	✓✓*	✓✓*	✓✓*	*S. carnaria*	*S. carnaria*
**14**	2002	✓✓	✓✓	✓✓*	✓✓*	✓	*S. teretirostris*	*S.emdeni*
**15**	2002	✓✓*	✓✓*	✓✓*	✓✓*	✓✓*	*S.variegata*	*S.lehmanni*
**16**	2002	✓✓*	✓✓*	✓✓	✓✓	✓✓*	*S.variegata*	*S.variegata*
**17**	2002	✓✓*	✓✓*	✓✓	✓✓*	✓✓*	*S.variegata*	*S.variegata*
**18**	2002	✓✓*	✓✓*	✓	✓	✓✓*	*S.lehmanni*	*S.variegata*
**19**	2003	✓✓*	✓✓*	✓✓	✓✓	✓✓*	*S.variegata*	*S.variegata*
**20**	2003	✓✓*	✓✓*	✓✓	✓✓	✓✓*	*S.variegata*	*S.lehmanni*
**21**	2006	✓✓*	✓	✓✓*	✓✓*	✓✓*	*S. carnaria*	*S. carnaria*
**22**	2007	✓✓	✓✓	✓✓	✓✓	✓	*S.subvicina*	*S.croatica*
**23**	2005	✓✓	✓✓	✓	✓	✓	*S.subvicina*	*S.croatica*
**24**	2015	✓✓*	✓✓*	✓✓	✓✓	✓✓*	*S.variegata*	*S.variegata*
**25**	2013	✓✓*	✓✓*	✓✓	✓✓	✓✓*	*S.variegata*	*S.variegata*
**26**	2013	✓✓*	✓✓	✓✓	✓✓	✓✓*	*S.variegata*	*S.variegata*
**28**	2011	X	✓✓	✓✓	✓✓	X	*S.crassipalpis*	*S.crassipalpis*
**29**	2011	X	✓✓	✓✓	✓✓	X	*S.crassipalpis*	*S.crassipalpis*
**30**	2011	✓✓*	✓✓	✓✓	✓✓	✓✓	*S.crassipalpis*	*S.crassipalpis*
**31**	2003	✓	✓✓	✓✓	✓✓	✓✓	*S.crassipalpis*	*S.crassipalpis*
**32**	2004	✓	✓✓	✓	✓	-	*S. argyrostoma*	*S.crassipalpis*
**33**	2004	✓✓	✓✓	✓	✓	-	*S.cultellata*	*S.crassipalpis*
**34**	2004	-	✓✓	✓	✓	-	*S. argyrostoma*	*S.crassipalpis*
**35**	2004	✓✓	✓✓	✓	✓	-	*S. argyrostoma*	*S.crassipalpis*
**36**	2004	✓	✓✓	✓✓	✓✓	-	*S. argyrostoma*	*S. argyrostoma*
**37**	2003	✓✓	✓✓	✓✓	✓✓	-	*S. argyrostoma*	*S. argyrostoma*
~~**39**~~	~~2006~~	-	-	-	-	-	-	~~*S.croatica*~~
~~**40**~~	~~2005~~	-	-	-	-	-	-	~~*S.croatica*~~
~~**41**~~	~~2016~~	-	-	-	-	-	-	~~*S.carnaria*~~
~~**42**~~	~~2002~~	-	-	-	-	-	-	~~*S.variegata*~~
**43**	2003	✓✓*	✓✓*	✓✓*	✓✓*	✓✓*	*S.variegata*	*S.lehmanni*
**44**	2002	✓✓*	✓✓	✓✓	✓✓	✓✓*	*S.variegata*	*S.variegata*
**45**	2002	✓✓*	✓	✓	✓	✓✓*	*S.variegata*	*S.lehmanni*
**46**	2002	✓✓	✓✓	-	-	✓✓*	*S.lehmanni*	*S.croatica*
**47**	2002	✓✓*	✓	-	-	✓✓*	*S.variegata*	*S.variegata*
**48**	2002	X	X	X	X	X		*S.variegata*
**49**	2002	X	X	X	X	X		*S.croatica*
**50**	2002	X	X	X	X	X		*S.variegata*
**51**	2004	X	X	X	X	X		*S.variegata*
**52**	2004	✓✓	✓✓	✓✓	✓✓	✓✓	*S.variegata*	*S.variegata*
**53**	2002	✓✓*	✓✓	✓✓	✓✓*		*S.variegata**	*S.variegata*
**54**	1983	✓✓*	✓	✓	✓		*S.carnaria**	*S.carnaria*
**55**	1983	✓	✓	✓✓*	✓		*S.variegata*	*S.subvicina*
**56**	1983	✓	✓	✓	✓		*S.variegata*	*S.carnaria*
**57**	1983	✓	✓	-	-		*S.variegata*	*S.subvicina*
~~**58**~~	~~2002~~	-	-	-	-	-	-	~~*S.variegata*~~
~~**59**~~	~~2002~~	-	-	-	-	-	-	~~*S.lehmanni*~~
**60**	1967	✓✓*	X	✓	✓			*S.carnaria*
**61**	1966	✓✓*	X	✓	✓✓*			*S.subvicina*
**62**	1946	✓✓*	X	✓✓*	✓✓*			*S.carnaria*
**63**	1942	X	X	X	✓			*S.carnaria*
~~**64**~~	~~2004~~	-	-	-	-	-	-	~~*S.carnaria*~~
~~**65**~~	~~2005~~	-	-	-	-	-	-	~~*S.croatica*~~
~~**66**~~	~~2005~~	-	-	-	-	-	-	~~*S.croatica*~~
**67**	1962		✓✓*	✓✓*	✓✓*		*S.carnaria**	*S.carnaria*
**68**	1948		X	X	X			*S.carnaria*
**69**	1977		-	-	✓✓*			*S.carnaria*
**70**	1977		✓✓*	✓✓*	✓✓*		*S.carnaria**	*S.carnaria*
**71**	1977		✓✓*	✓✓*	✓✓*		*S.carnaria**	*S.carnaria*

**72**	1971		✓✓*	✓✓*	✓✓*		*S.carnaria**	*S.carnaria*
**73**	1954		✓✓*	✓✓*	✓✓*		*S.carnaria**	*S.carnaria*
**74**	1954		✓✓*	✓✓*	✓✓*		*S.carnaria**	*S.carnaria*
**75**	1953		✓✓*	✓✓*	✓✓*		*S.carnaria**	*S.carnaria*
**76**	1951	X	-	-	✓	X		*S.carnaria*
**77**	1932	X	-	✓✓*	✓	X	*S.carnaria**	*S.carnaria*
**78**	1931		-	✓✓*	X		*S.carnaria**	*S.carnaria*
**79**	1930	*X*	*X*	-	✓	*X*	*S.carnaria**	*S.carnaria*
**80**	1936	*X*	-	✓	*X*	*X*		*S.carnaria*
**81**	1920	*X*	*X*	✓✓*	✓	*X*	*S.albiceps*	*S.carnaria*
**82**	1919	*X*	-	-	✓	*X*		*S.carnaria*
**83**	1902	*X*	-	-	✓	*X*	*S. carnaria*	*S.subvicina*
**84**	1898	*X*	*X*	-	✓	*X*		*S.subvicina*
